# Broth Optical Density-Based Assessment for Phage Therapy: Turbidity Reduction, Antibacterial Virulence, and Time-Kill

**DOI:** 10.3390/v18010097

**Published:** 2026-01-10

**Authors:** Stephen T. Abedon

**Affiliations:** Department of Microbiology, The Ohio State University, Mansfield, OH 44906, USA; abedon.1@osu.edu

**Keywords:** 96-well plate, bacterial growth reduction, bacteriophage, bacteriophage therapy, kinetic assay, lysis profile, optical density, time-kill, time-killing, time kill

## Abstract

Phage therapy is the use of bacterial viruses, or bacteriophages, as antibacterial agents. It has been in use for over 100 years and is becoming increasingly common clinically. The first steps of phage therapy include identification of bacteria to be targeted and then obtaining phages with appropriate host ranges. This is followed by various approaches to in vitro phage characterization. Increasingly common for phage phenotypic characterization is the use of kinetic microtiter plate readers. They can both decrease workloads and increase throughput, especially relative to analyses that require plating on agar-based media. These colorimetric/turbidimetric/optical density approaches primarily assess phage-induced culture-wide bacterial lysis, in the shorter term, or instead the phage potential to suppress phage-resistance evolution over longer time frames. Considered here are methods relevant to phage characterization especially for phage-therapy purposes. Discussed are turbidity-reduction assays, determinations of phage antibacterial virulence, and related time-kill curve analysis. All are or can be optical density-based approaches to assessing phage-based bacterial reduction. Emphasis is placed on consideration of the utilities, limitations, and intersections of these similar methods. Emphasized is that the start of “Deviation”—where phage-treated culture turbidity diverges from phage-free controls—may represent a superior endpoint for such optical density-based bacterial-reduction protocols.

## 1. Introduction

The use of bacteriophages (phages) as antibacterial agents dates back to the late 1910s [1,2], with the first official publication on clinical phage therapy dating from 1921 [3,4]. The key to successful phage therapy is pairing a targeted bacterium with a phage that is able to kill that bacterium. Principally, this involves reducing bacterial numbers within a treated environment to a sufficient extent while preventing substantial grow back of resistant bacteria. While in vivo environments provide a better test of antibacterial effectiveness, the difficulty of treating whole organisms—including the expense and ethical issues associated with animal testing—necessitates first testing phages in vitro for antibacterial activity.

Complicating in vitro testing, at least four levels of phage antibacterial activity may be characterized for a given, targeted bacterial strain. These include (i) a phage’s ability to replicate to higher numbers in situ during treatments (so-called auto dosing [5]), (ii) a phage’s ability to kill bacteria (bactericidal activity), (iii) a phage’s ability to lyse bacteria (bacteriolytic activity), and (iv) a phage’s ability to reduce bacterial evolution of phage resistance, as can result in culture grow back [6,7]. Emphasis here is on the first three of these abilities.

Many aspects of phage antibacterial activities can be tested using solid and semisolid media. Spot testing, for instance, can be performed employing high titers of phages, which can serve as a test of bactericidal activity [8,9]. Alternatively, using dilution series and thereby lower titers will promote the formation of individual plaques [10,11]. Plaque formation is dependent for most phages on a combination of phage-induced bacterial lysis and associated phage population growth. Taken together, these two properties in a phage-therapy context make up auto dosing [5] (though not an emphasis of this review, chronically infecting phages also can form plaques and auto dose, but without lysing their bacterial hosts [12]). Furthermore, by supplying sufficient numbers of phages to result in confluent lysis of bacteria—across a lawn [13,14], within a spot, or even within a plaque [15]—it is possible to observe bacterial development of phage resistance. This can be seen in the form of isolated bacterial colonies surrounded by a cleared bacterial lawn.

Petri dish-based methods are well established, but they also are relatively labor- and materials-intensive, particularly when multiple types of phages are being tested against multiple types of bacteria. To improve throughput, one can alternatively employ broth cultures to explore various phage phenotypic characteristics. In particular, it is increasingly common to assess the phage impact on bacteria using optical-density methods. This can include (1) determining phage viable counts (i.e., titers [16]) in terms of reductions in culture turbidities, (2) the potential for phages to induce culture-wide lysis of bacterial cultures, (3) the timing of that lysis, (4) phage host-range determination, and (5) the potential for bacteria to mutate to phage resistance. All of these approaches can be accomplished, for example, within 96-well microtiter plates and automated using kinetic-reading microtiter plate readers, as I have previously reviewed [17].

Considered in this review is how to improve upon these and other in vitro approaches to phage characterization. An emphasis is placed on the potential utility of the phenomenon dubbed here as “Deviation” relative to other optical-density milestones in phage population growth (Section 2). Deviation is the point in optical-density curves where the turbidity of a phage-containing culture diverges from that of the phage-free bacterial control [16,17,18,19,20]. Use of this deviation metric helps address a general limitation to the use of turbidity as an approach to determining phage suitability for phage therapy purposes, which commonly emphasize the timing of culture-wide phage-induced bacterial lysis. Specifically, the timing of that lysis occurs well after the point at which phages have infected a large fraction of bacteria, i.e., after roughly one latent period. Phage bactericidal activity, however, occurs instead at the point of phage adsorption to bacterial cells—the start of the phage latent period—which can be quantified as reductions in colony-forming units (CFUs). The point where a large fraction of bacteria have become phage-infected can roughly coincide with the point of turbidity deviation during optical-density assays (S.T.A., unpublished observation).

Often for turbidity-based approaches to phage characterization, both deviation and subsequent phage-induced bacterial lysis are preceded by phage population growth. Though phage population growth is required for what can be described as active treatment [5,21,22], it is not necessarily always a concern with clinical phage therapy protocols, especially if phages can be delivered to bacterial targets in high numbers using traditional approaches to dosing (i.e., passive treatments). Thus, a typical optical density-based phage-characterization experiment involves phage replication to higher numbers, phage bactericidal activity (coinciding more or less with deviation), and then phage bacteriolytic activity. This is rather than applying higher concentrations of phages from the start and thus skipping the need for phage replication to higher numbers.

This sequence, when followed using optical density means, can be described as a turbidity reduction assay, which in turn is often considered to stem from reductions in bacterial numbers (Section 3). Phage antibacterial-virulence assays represent a variation of bacterial reduction experiments, focusing especially on what starting phage numbers or ratios to bacteria are necessary to result in culture-wide bacterial lysis, including in terms of its timing (Section 4). Phage antibacterial virulence assays in turn have parallels with what in the antibiotics literature are dubbed as time-kill curves (Section 5) and which here I suggest can be more efficiently generated for phages based on culture optical density rather than the traditional CFU-based methodology. Relevant to that suggestion, deviation should provide a better approximation of the timing of bacterial killing—the “kill” of time-kill curves—than the timing of phage-induced bacterial lysis.

The central arguments of this review thus are that (1) there exist multiple related optical density-based approaches that researchers have developed to assess the antibacterial impact of phages on bacteria (turbidity reduction, antibacterial virulence, and also variations of time-kill curves), (2) that optical density-based phage characterization assays generally should be measuring bacterial reduction (killing) rather than solely turbidity reduction (lysis), and (3) that the deviation metric provides a means to assess bacterial killing using optical density approaches. The progression of the review is an introduction to quantifying phage impact on culture optical density (Section 2), a look at the traditional generic end point (turbidity reduction; Section 3), a more sophisticated approach to comparing phages (antibacterial virulence; Section 4), an even better approach but one that is much more labor intensive (time-kill curves; Section 5), and then making “Better” less labor intensive via introduction of deviation as a correlate of time killing (also Section 5).

Further optical density-based methods useful for therapeutic phage characterization exist, but are beyond the scope of this review. These include checkerboard assays (e.g., [23,24]) and related determinations of phage–antibiotic synergy (PAS) [25,26,27,28,29,30,31,32], along with testing for antibiotic-mediated antagonism of phage infection activities [33,34]. Bacterial evolution of phage resistance [6,17], though mentioned, is not an emphasis of the review.

## 2. Optical-Density Milestones in Phage Population Growth

**Figure 1:**
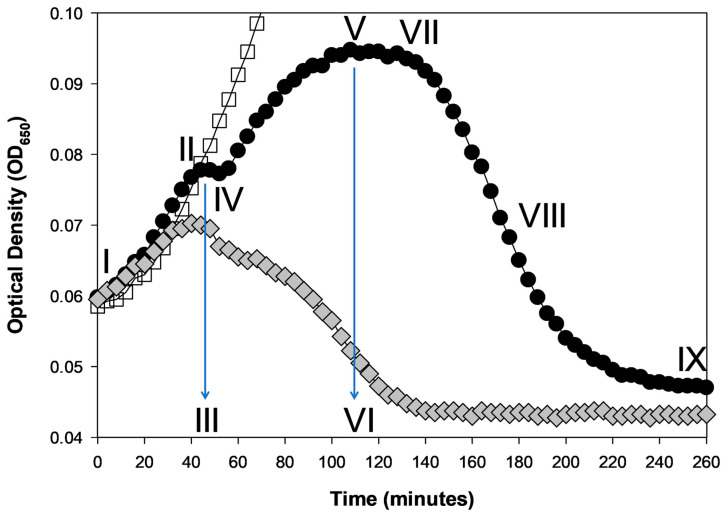
Features of a lysis profile. Shown are phages LZ3 (dark circles, ●) and K3 (gray diamonds, ♦), infecting *Escherichia coli* B (or without phage infection, open squares, ☐). Both phages may be displaying lysis inhibition, as I infer based on the shape of their curves. The resulting extended latent periods, especially as seen here with phage LZ3, allow for labeling with less clutter (without lysis inhibition, lysis here would be expected to have been completed by around 60 min for both phages). So too the *y* axis has been truncated at a maximum optical density of 0.1 (roughly 10^8^ bacteria/mL) so as to emphasize in the graph the phage-containing curves (● and ♦) rather than phage-free bacterial growth (☐). Roman numerals label the phage LZ3 curve (●) and correspond to (I) starting turbidity, (II) start of deviation (where most bacteria are now presumably phage-infected), (III) timing of the start of deviation (as indicated with the arrow), (IV) lysis of a portion of phage-infected bacteria (or an adsorption-associated turbidity drop), (V) peak culture turbidity (OD_max_), (VI) timing of that peak (also as indicated with an arrow), (VII) time of start of substantial lysis (though this is not always straightforward to define), (VIII) duration of that lysis, and (IX) time of the end of the turbidity decline (time to extinction). Not indicated in this figure though discussed in the main text is (X), regrowth of cultures associated with bacterial resistance (see the following figure for illustration of this). Phages were added at a multiplicity of somewhat less than 1 at time 0. This allowed the bacterial culture to increase in turbidity uninhibited until multistep phage population growth caught up numerically with that of the bacteria. The host is *E. coli* B and the growth medium is trypticase soy broth (TSB) supplemented with 2.9 g/liter NaCl. The experiment was run using a Molecular Devices Thermomax microtiter plate reader that was set to 37 °C, 3 s of shaking before each time point, and with time points taken every four minutes using a 650 nm filter. Shown are averages of three technical replicates of a single biological replicate. See [17] for further discussion of this protocol.

The following milestones may be observed in the course of phage population growth using optical density measures, that is, for lysis profiles (Figure 1) [35,36,37,38]:

(I) Starting culture turbidities;
(II) Culture turbidities at the start of curve deviation from that of the phage-free control;
(III) Timing of the start of deviation;
(IV) Temporary pauses or declines in culture turbidities in the presence of certain phage types [39,40,41] (particularly those that can display lysis inhibition, which is a latent period extension displayed by certain phages that is induced by phage secondary adsorption [15,16,17] and which is illustrated in Figure 1 especially by the phage LZ3 curve);
(V) Peaks of culture turbidity in the presence of phages (OD_max_);
(VI) Timing of OD_max_;
(VII) Timing of the start of substantial declines in culture turbidities [42]; and
(VIII) Overall durations of these culture declines in turbidity;
(IX) Time of the end of turbidity declines [16].

In addition (X), though not indicated in Figure 1 (but see the next figure, below), increases in culture turbidity may then be observed that are thought to correspond to growth of phage-resistant bacteria [17]. Though bacteria can in some cases develop a tolerance for phages instead [43], here these post-lysis rises in culture turbidity are referred to as being due simply to genetic phage resistance.

Typically, in terms of phage impact on bacteria, interest is in turbidity reductions (Section 3.1), i.e., as indicated by VII and VIII in Figure 1. This is rather than observations of only less rapid rises in turbidities (Section 3.2), as researchers often are interested in more concrete evidence especially of phage-induced bacterial lysis. Indeed, assessing for substantial reductions in culture turbidities seems to represent a majority of uses of optical density measures for phage therapy purposes. This involves adding phages to bacterial cultures and then looking for what ultimately should be an absence of discernible turbidity. But how best to quantify that bacteriolytic event, or any other phage impact?

A very early example of the use of changes in culture turbidity to quantify the impact of phages on bacteria is the work of Krueger, from 1930 [42]. There, the timing of culture-wide bacterial lysis, essentially the decline associated with VIII in Figure 1, was correlated with starting phage titers. A simpler way of obtaining similar information involves first determining the point of peak culture turbidity (OD_max_) and then assessing its timing (OD_max_ timing) [44]. That is, by definition declines in culture turbidity will follow those peaks (V in Figure 1), even if subsequent declines are slight. Especially for phages that display lysis inhibition or other delays in phage-induced bacterial lysis, OD_max_ timing nevertheless can be far removed from when lysis occurs in earnest (V vs. VII and VIII in Figure 1; Section 4.3 and Section 4.4). Thus, while convenient as a measure of the start of phage-induced culture-wide bacterial lysis, the timing of OD_max_ can be an imperfect predictor of the timing of substantial reductions in culture turbidities.

The actual peak turbidity of cultures (simply OD_max_) too can be used as a measure of the impact of phages on bacteria [45]. An alternative approach is to measure the timing of the end of lysis [46], though I suspect that is a method that is less useful when comparing different phage types rather than different genotypic variants of the same phage isolates. Also useful is the above-noted timing of the start of deviation of phage-containing curves from those of phage-less cultures (point III, Figure 1; Section 2.1). For recent analyses of the utility of these different approaches to quantifying lysis profiles, see [16,47].

### 2.1. Utility of Deviation and Especially the Timing of Its Start

In many ways, the timing (III) or turbidity (II) at the start of deviation of a phage-containing culture is a more complicated metric to objectively obtain than others indicated in Figure 1. This difficulty stems especially from random variation that can often be seen between concurrently generated optical density curves: Thus, when specifically does the start of deviation occur? In addition, the precision of this measurement is a function of how close together time points are taken. Consequently, 1 h time intervals, as are often observed when assays are run over many hours (e.g., see Appendix A), provide little information as to the specific timing or turbidity of the start of deviation. Despite these concerns, the precise start of deviation in principle can be determined objectively using computational methods (S.T.A., in development).

**Figure 2:**
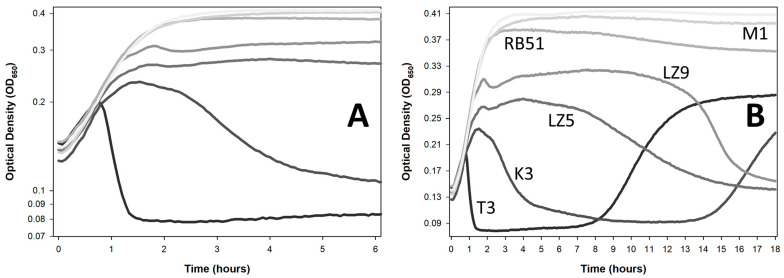
Turbidity reduction assays. Both figures are based on the same optical-density data with different y-axis scales: (**A**) logarithmic scale; (**B**) linear scale. The names of the different phages are indicated in panel B, with the top and lightest curve corresponding to the phage-free control (line shades otherwise are consistent between the two panels). Averages of three technical replicates are shown with all curves generated during the same experiment. All phages were applied with multiplicities of somewhat less than 1, though actual multiplicities were not otherwise controlled for. Phages T3 and K3 show unambiguous turbidity reductions, as well as grow back of presumptively phage-resistant bacteria. Phages T3, K3, LZ5, LZ9, and RB51 all show an OD_max_, which is strongly suggestive of actual bacterial reductions even though reductions were never to the point of below that of starting turbidities (an OD of around 0.13), except for phages T3 and K3, both of which also showed the earliest and most robust lysis. Phage M1 may or may not be having any impact. The experiment otherwise was run equivalently to that shown in Figure 1.

This start of deviation—II and III of Figure 1—likely represents the closest optical density-based correlate to bactericidal impact on a bacterial population [16] (Section 5 but also Section 4.5). Specifically, the sooner that deviation occurs, likely the faster the phage population has substantially reduced the bacterial population in viability. Qualifications to that statement, however, include the following:

- This assumes that one is working with obligately lytic phages, so that virion impact means bacterial adsorption that leads directly to bacterial killing;
- This assumes that culture turbidities do not continue to rise, without deviation, even given substantial phage adsorption and infection, as that would represent a false indication of bacterial survival (e.g., it is uncertain when exactly a majority of bacteria became infected especially in the RB51 curve in Figure 2); and
- This assumes that deviation does not represent just a pause in turbidity rise that is followed by substantial reduction to lysogeny (i.e., prophage formation), which would also represent a false indication of bacterial killing (though to the extent that temperate phages are avoided, this should be less of an issue for phage characterization for phage therapy purposes).

Nonetheless, the lower the turbidity at which deviation occurs for a given treatment protocol then the fewer bacteria that have been allowed to be present prior to becoming phage-infected. This should hold even if observable deviation begins later than the point of substantial bacterial adsorption and infection (as noted in the second qualification, above).

The start of deviation may be observed as only a reduction in turbidity growth rather than an actual reduction in culture turbidity (slowed increase rather than an actual decrease). Nonetheless, if the goal of optical density assays is to measure a phage’s negative impact on bacterial viability, then the point of deviation likely represents the best correlate for that occurrence. While calculation of the timing of deviation and correlation of its occurrence with substantial bacterial killing require further validation and refinement, initial experiments are encouraging (S.T.A. unpublished observations).

### 2.2. Areas Under Curves

In addition to the metrics discussed in the previous section, area under the curve (AUC) determinations are often used to compare lysis profile results, e.g., [48,49,50,51]. AUCs are convenient. They supply a single number that indicates total amounts of bacterial biomass, consisting especially of intact bacterial cells that are present over some interval of time. This section, however, considers a number of potential shortcomings to AUC-based determinations of bacterial reduction.

#### 2.2.1. AUCs Are Complex and Do Not Directly Measure Bacterial Reductions in Viability

AUCs are composite measures of phage impacts on bacteria. Generally speaking, AUC magnitudes thus tend to be bigger

- The larger starting bacterial concentrations;
- The greater the delay until a majority of bacteria become phage-infected;
- The longer phage latent periods;
- The slower the process of culture-wide bacterial lysis once begun;
- The higher the turbidity following the completion of lysis, and;
- The more that phage-resistant bacteria contribute to AUC calculations.

None of these measures directly describe the extent to which bacterial viability across a culture is reduced over time by a phage population. Consequently, if the question specifically is how rapidly different phages impact the ability of bacteria to continue to reproduce—that is, as bacterial viability reduction assays (Section 5)—then AUC-based determinations may not be ideal.

#### 2.2.2. AUCs and the Non-Equivalence of Turbidities

In addition to the issues considered immediately above, culture turbidity also cannot on its own distinguish bacteria that are phage-infected from bacteria that are not phage-infected. Additionally, turbidity cannot distinguish phage-sensitive but uninfected bacteria from phage-resistant bacteria. Though inclusion of these different categories of bacteria is not necessarily always problematic, it is useful nonetheless to appreciate that this ambiguity exists, and to understand its impact on optical density-based conclusions.

Differentiating phage-infected from phage-uninfected bacteria is crucial to the extent that turbidity reductions are being used to track reductions in bacterial viability. This is because phage-infected bacteria can still be physically intact despite no longer being able to produce new bacteria (Section 4.3 and Section 4.4). Furthermore, evolution of phage-resistant bacteria during experiments is mostly independent of considerations of either phage host range or phage-antibacterial virulence ([17] and Section 4 for the latter). Yet, both of those latter properties—phage host range and phage antibacterial virulence—are crucial determinants toward achieving initial bacterial reductions during phage therapies: the elimination of phage-sensitive bacteria.

This issue of distinguishing infected from uninfected bacteria and sensitive from resistant bacteria can also be seen with non-AUC metrics of phage impact (Figure 1). Importantly, though, some distinctions can be made via more detailed curve observations that cannot be made based solely on AUC calculations. For instance, the presumed transition from mostly uninfected to mostly phage-infected bacteria during experiments likely occurs at the start of deviation of phage-present from phage-absent curves (II, Figure 1). So too, the transition to substantial numbers of phage-resistant bacteria can be seen as a change in the trajectory of curves from constant or declining to instead rising. The latter, however, can also be avoided by simply not including later time points in AUC calculations [50] or by not allowing phage-sensitive bacteria to ever grow to higher numbers [17].

Distinguishing especially phage-infected from not-infected bacteria is less readily accomplished based solely on AUC determinations. To the extent that phage infections are bactericidal—as they ought to be when employing obligately lytic treatment phages [52]—then this transition is especially relevant to the employment of turbidity reduction assays as a means of assessing phage-mediated bacterial reductions. Thus, AUC determinations take into account far more aspects of phage-bacterial interactions than any one of the measurements indicated in Figure 1. Furthermore, and importantly, those different aspects have different implications regarding phage suitability for phage therapy purposes—different implications that ideally would be considered separately rather than being lumped into a single metric.

#### 2.2.3. Those and Other AUC Issues

Provided in this section is a summary of these and additional issues that can be relevant to the usefulness of AUC-based optical-density analyses of phage impacts on bacteria. They include:

- After culture-wide bacterial lysis, AUCs can be confounded by the regrowth of bacterial cultures that can occur due to the presence of phage-resistant bacteria, whether bacterial mutants or newly formed bacterial lysogens, and this is particularly if assays are run for too many hours post-lysis, e.g., [17,48,53,54,55,56].
- Phage display of lysis inhibition or other mechanisms of latent period extension [17] will inherently result in greater areas under curves (Figure 1) due to a vast majority of bacteria remaining lytically phage-infected over much of the course of experiments (delaying the start of turbidity declines) and/or displaying slower turbidity declines once lysis begins (Section 4.3 and Section 4.4).
- Uninfected bacteria do not necessarily remain constant in size and thereby constant in biomass over the course of growth to higher numbers, especially with an expectation that sizes of individual bacteria will decline as bacteria approach stationary phase [57].
- Phage-infected bacteria can increase in size over time [58,59], resulting in greater optical density with the same number of intact but nonetheless no longer reproductively viable bacterial cells.
- For optical density measures to accurately reflect bacterial biomass, individual bacteria need to be randomly dispersed rather than clumped (this is a larger issue with certain bacteria than with others, such as with *Staphylococcus* [60], but also with *Pseudomonas aeruginosa* [61]). This and the three previous also can affect OD_max_.
- Following culture-wide bacterial lysis, cultures often retain a greater turbidity than that of sterile broth or even a greater turbidity than that associated with starting bacterial concentrations (Figure 2), thereby non-specifically enhancing AUC values.
- The ratios of AUCs between different phage types will tend to vary depending on the interval over which curves are integrated, that is, started and then stopped (described as limit selection [62]), and this can be true even independent of phage resistance evolution.
- Though not technically a shortcoming explicitly of AUC determinations, it is important for authors to indicate within a study over what timeframes AUC integrations have been made.

Given this plethora of potentially complicating issues associated with the use of AUC measures of bacterial turbidity reduction, researchers should at least consider alternative approaches toward quantifying the phage impact on bacterial cultures (Figure 1).

### 2.3. Centroid Determination

Hosseini et al. [63] provide a mathematical alternative to AUC calculations, called “center of area (centroid)”. This approach, however, suffers from many of the biological shortcomings of AUC calculations (Section 2.2.3). This is not to say that AUC or centroid calculations are devoid of utility. Nonetheless, they have numerous confounding aspects that serve to limit their potential to quantify phage-mediated bacterial reduction—particularly as may be detected based on changes to bacterial viability. This general issue in fact was explicitly stated by Hosseini et al. [63]: “The centroid index or any other available indices may not capture all the detailed fluctuations of a growth curve and therefore depending solely on these indices may overlook subtle nuances in phage behavior.”

### 2.4. Monitoring Lysis Using Metabolic Indicators

An alternative approach to visualizing phage-induced, culture-wide bacterial lysis, i.e., as an indication of bacterial reduction, is to follow this lysis using metabolic indicators. This is consistent with the theme here of considering optical density or absorbance measures of planktonic bacterial presence. Emphasis, however, is placed instead on the use of dyes, specifically tetrazolium dyes, which indicate both current and prior presence of intact, metabolizing bacteria (standard optical-density approaches predominantly indicate just current presence of intact bacteria). Henry et al. [64] appear to have pioneered this approach for the study of phage infections of planktonic bacteria.

Though innovative, the approach—like optical density-based approaches generally—cannot readily distinguish phage-infected but still-metabolizing bacteria from phage-uninfected bacteria. This, I speculate, should make it more difficult to characterize the virulence of phages (Section 4) that display lysis inhibition (Section 4.3), or other delays in lysis (Section 4.4). Such delays in lysis, that is, should allow ongoing, i.e., hours-long bacterial metabolism despite their representing bactericidal phage infections. So too this is an issue for turbidity-based approaches, though such delays affect AUC or OD_max_ determinations more than the start of deviation. Importantly, with metabolic indicators, continued bacterial cell division should be readily distinguishable from an absence of cell division, thereby providing a potential for precise determination of the start of deviation.

## 3. Turbidity Reduction Assays

Most of this review considers phage impacts on the turbidity of bacterial cultures. This primarily involves decreases in those turbidities such as due to phage-induced bacterial lysis—thus, turbidity reduction. Typically such reductions in culture turbidities are thought to correlate with declines in numbers of physically intact bacteria. Thus, even though based on changes in culture turbidities, resulting assays often are referenced in terms of bacterial reduction. However, phage infection of individual bacteria precedes—in some cases by many hours [15,16,17]—phage-mediated bacterial lysis. This temporal discrepancy is an issue returned to throughout this review.

Synonyms for determinations of reductions in bacterial numbers or reductions in bacterial culture turbidities are numerous. They include antibacterial activity, bactericidal activity, challenge assay, effect on growth curves, efficacy test, inactivation, killing assay, killing efficacy, lysis assay, lysis kinetics, lytic ability, lytic capacity, phage susceptibility, planktonic killing assay, time to extinction, etc.

Perhaps the simplest and probably oldest [65] approach to phage characterization, in terms of culture optical density, is what can be dubbed a “Bacterial reduction assay” [66] or “Bacterial growth reduction assay” [67,68,69,70]. In this section, those two concepts, taken literally, are contrasted.

### 3.1. Bacterial Reduction

“Bacterial reduction” assays consist of the application of phages to bacterial cultures that are then followed by observation for drops in bacterial presence. Such assays may require increases in phage numbers to effect these reductions, as equivalent to active treatments. Alternatively, they may not require those increases, should initial numbers of phages exceed those of bacteria, which effectively is a passive treatment. Note, though, that it is possible for even successfully adsorbing phages to fail to substantially lyse a bacterial culture. This can occur if those phages are added at insufficiently high numbers, adsorb too slowly, otherwise grow their populations slowly, or have difficulty lysing bacteria found at higher concentrations [17]. The latter can be bacterial concentrations found upon phage application or instead as reached in the course of bacterial (and phage) population growth. All of those factors also can impact determinations of phage antibacterial virulence as that virulence is often assayed (Section 4).

An important feature of such reductions during kinetic assays is peak turbidity, i.e., a turbidity that subsequently declines. If a peak in optical density is seen, then that can serve as a convenient way to quantify phage impact on a bacterial culture; that is, based on peak optical density magnitude (OD_max_) or instead its timing (OD_max_ timing) [16,45,47]. An OD_max_ furthermore may be seen even if ending culture turbidities are not lower than starting turbidities, as can occur (1) if phage-induced lysis is inefficient in reducing culture turbidities to those seen with sterile broth and (2) starting bacterial concentrations are relatively low. An OD_max_ that is followed by reductions in culture turbidities is suggestive, nonetheless, of phage-induced bacterial lysis, and particularly so if the resulting turbidity-drop is substantial. Thus, in a broader rather than strict sense, a turbidity reduction may be characterized as a phage impact that results in a peak in culture turbidity that is followed especially by a substantial drop in culture turbidity. That substantial drop, however, does not necessarily immediately follow OD_max_ for all phages under all circumstances (e.g., Figure 1).

“Substantial” is unfortunately left ambiguous. Ideally, it would be at least to below whatever culture turbidity was reached at the point where a phage-containing curve’s turbidity deviates (becomes lower) than the turbidity of a phage-less culture control. See Figure 2 for examples. There, all but the phage M1 and phage RB51 curves appear to display unambiguous turbidity reductions, though not all reductions are to below the starting turbidity nor even to below the point of deviation (phages LZ5 and LZ9). Furthermore, there exists variation in the rapidity of those turbidity reductions. Thus, what actually represents a bacterial reduction as based on optical density information may be more obvious for some phages, or circumstances, than for others. Subsequently, however, I argue that this lysis event may not actually be the best indicator of bacterial reduction.

### 3.2. Bacterial Growth Reduction

Alternatively, there is bacterial *growth* reduction. Again, if taken literally, this would differ from strictly bacterial reduction in that culture turbidity and thereby bacterial numbers need not decline to a noticeable extent. Thus, the defining feature of bacterial growth reduction can be a turbidity endpoint that is lower when phages are present than without phages present, though substantial reductions in turbidity are not necessarily observed. For such an assay, starting culture turbidities could be fairly low and then scored positive based on a failure to substantially increase in turbidity, such as may be monitored simply by eye in a most probable numbers-type assay [17].

Just as with actual turbidity reductions (Section 3.1), with kinetic assays, bacterial growth reductions should start with deviation of the optical density of phage-containing cultures away from that of the phage-lacking control. Thus, the culture that contains phages comes to display a lower turbidity than the still-replicating phage-free control, but without actual or substantial drops in turbidity. The culture instead stops increasing in turbidity at the same rate as the phage-less control.

It is at the start of this deviation that turbidity increases begin to slow, which likely corresponds to phage infection of a relatively large fraction of those bacteria present (S.T.A., unpublished observation). Whether just slowing is due to phage adsorption of only some smaller fraction of bacteria, however, requires experimental verification, such as in terms of plating for still-viable bacteria (Section 5.2). Specifically, it is conceivable that the start of deviation marks a point at which a large majority of bacteria have come to be phage-infected—rather than some smaller fraction—but with the culture nevertheless continuing to increase in turbidity despite a lack of actual bacterial replication [58,59]. Thus, the mechanisms underlying phage-mediated bacterial growth reduction are likely less straightforward than simple bacterial reduction as indicated by substantial phage-mediated drops in culture turbidities.

See Figure 2 for example curves. All employ starting phage multiplicities of somewhat less than one, and in most cases deviation appears to be present. Indeed, phage RB51 exemplifies a curve where deviation seems to occur but without a substantial turbidity decline over the course of the presented 18 h incubation. This also may be true for the phage M1 but with even less turbidity reduction.

### 3.3. Absolute Reductions Generally Are Preferable

In practice, these two phrases—bacterial reduction and bacterial growth reduction—seem to be used largely interchangeably. There nevertheless are certainly examples of phages appearing to slow bacterial growth without giving rise to an obvious or extensive culture-wide lysis event [19,48,50,71,72,73,74,75,76]; see also Figure 2, phage RB51 as well as phage M1. This seeming prevention of turbidity rises without obviously lysing, however, can be due to methodological artifacts [16]. Those can include the following:

- A need for *y*-axis expansion to better visualize lytic events;
- Starting with excessively high phage multiplicities in combination with excessively low bacterial concentrations, resulting in turbidities that are too low to easily detect changes; and
- Insufficient duration of incubations to observe an eventual reduction event (Section 4.3 and Section 4.4).

In addition, growth of phage-resistant bacteria can obscure lysis-associated turbidity declines. Even when phages do not give rise to an obvious turbidity-reduction event, it is likely, as noted, that slowing of bacterial “growth” actually corresponds to phage infection of at least a non-trivial fraction of bacteria present. Whether that assertion is true for all phages under all circumstances, however, remains to be seen.

Thus, though there clearly exists an observable difference between obligately lytic phages causing a reduction in culture turbidity (bacterial reduction assay, sensu stricto) vs. just slowing down the rate of increase in turbidity (bacterial growth reduction assay, sensu stricto), in practice little difference in terms of resulting bacterial viability may exist. Exception to that claim, however, could occur if working with phages that are bacteriolytic but unable to produce virions. In that case, it is possible to run out of treatment phages within cultures as those limited numbers of phages adsorb bacteria, a concept that can be described as a consequence of sorptive scavenging [43,77,78]. That is, without phage in situ replication, a slowing of bacterial growth could in principle be followed by a recovery of phage-sensitive bacteria.

If there is doubt about bacterial viability, then one should plate for bacterial survival (Section 5), and do so soon after deviation is observed. That timing is because if plating is performed later, then phage-resistant bacteria could come to dominate bacterial populations, obscuring the phage impact on phage-sensitive bacteria. Indeed the primary goal of a bacterial-reduction or turbidity-reduction assay should be to quantify reductions in phage-sensitive bacteria, rather than also taking into account subsequent, substantial increases in phage-resistant bacteria [17].

### 3.4. Turbidity Reduction as a Foundation for Related Approaches

Turbidity reduction represents a foundational concept that can be elaborated upon in various ways. The basic approach—observing phage-induced declines in culture optical density—can be refined to assess phage antibacterial virulence through systematic variation of starting conditions (Section 4). Additionally, the concept can be extended to time-kill assays, which traditionally measure bacterial reduction as CFU declines but which may also be approximated using optical density means (Section 5). An important consideration throughout is that even subtle changes in turbidity can provide substantial information. Specifically, the point of deviation (Section 2.1) could represent a means of capturing bacterial killing before the more dramatic turbidity decline associated with culture-wide bacterial lysis begins.

## 4. Phage Antibacterial Virulence

“…the important thing is the quality of the bacteriophage, that is, its virulence.” D’Herelle [65], p. 68 (from 1922).

While ‘virulence’ in broader microbiology typically describes a pathogen’s capacity to cause disease in a host organism, in phage biology it has historically referred to multiple distinct properties [79,80,81]. Perhaps the most common meaning within the phage therapy literature is that of phage antibacterial virulence [16,48,50,51,65,82,83,84], which is the definition employed here. That is, a phage’s ability to bring especially phage-sensitive bacteria under control by killing those bacteria, while not also taking into account bacterial evolution of phage resistance [17,50] (potentially, see also [65,82]). For both of those reasons—emphasizing killing while deemphasizing resistance evolution—I therefore again point to the start-of-deviation metric (Section 2.1) as possibly superior to other means of measuring that phage impact using optical density means; in this case toward assessing a phage’s antibacterial virulence. Ideally, deviation signifies the phage infection and thereby genetic death of a majority of bacteria within a culture, with that bacterial reduction (Section 3) typically occurring many hours prior to resistance evolution becoming an optical-density issue.

### 4.1. Digging Deeper into the Meaning of Phage Antibacterial Virulence

Phage antibacterial virulence assays represent an elaboration of the bacterial reduction assays discussed in Section 3, quantifying how efficiently phages kill bacteria under varying conditions. With bacterial reduction assays, the question being asked is, can a given phage negatively impact bacterial presence? The answer to that question can be binary, i.e., either yes or no. As noted, however, there can be degrees of impact, which in Section 3 are distinguished into bacterial reduction vs. bacterial growth reduction. What a phage antibacterial virulence assay attempts to accomplish is to quantify those distinctions as well as to explore the impact of varying starting ratios and quantities of phages and bacteria. Thus, a phage antibacterial virulence assay can be viewed as an approach to more fully explore and quantify turbidity reduction.

D’Herelle [65], in his 1922 monograph, “*The Bacteriophage: Its Role in Immunity*”, defined phage virulence as an “ability to multiply at the expense of the parasitized being” (p. 27) and suggested that “each strain of bacteriophage is endowed with an individual degree of virulence”. On the same page, he went on to note that, “Certain races of the bacteriophage multiply rapidly, others increase but slowly. The first possess a high degree of virulence toward the bacterium provided for their development; the second possess but a feeble virulence.” It is distinguishing between this “high” and “feeble” virulence that a modern phage antibacterial virulence assay attempts to achieve.

D’Herelle [65] then goes on to describe virulence as a phage property that can give rise to culture lysis despite starting with higher numbers of bacteria (p. 31): “I might cite as an example an anti-staphylococcic strain with which Eliava was forced to make passages during four months in order to obtain sufficient virulence to induce complete lysis of a suspension containing 500 million staphylococci per cubic centimeter.” In addition, from p. 66, he considers “a bacteriophage of maximum virulence before which the bacteria always succumb.” Similarly (p. 69), “The intensity of the action upon a bacterial suspension, or on a culture, in a liquid medium gives an indication of the virulence.” Thus, d’Herelle described (1) a concept of phage antibacterial virulence, (2) the potential for that virulence to vary between different phage types, (3) a potential for phages to change in their virulence with repeated passage, and (4) the use of the phage impact on bacterial culture turbidity as a means of distinguishing phages in terms of their antibacterial virulence. For the latter (p. 69), he means “to measure their respective powers of growth at the expense of the bacteria parasitized, that is to say, their virulence.”

Smith and Huggins [82], in 1983, appear to have expanded on d’Herelle’s ideas on phage antibacterial virulence. They wrote (p. 2661), “Judged on the number of phage particles required to lyse bacterial cultures, phage B44/1 was more virulent for *E. coli* B44 than phage B44/2 or, especially, phage B44/3; phage B44/3, though, was much more virulent than phage B44/2 for mutants of *E. coli* B44 resistant to phage B44/1. Phage P433/1 was highly virulent for *E. coli* P433 and phage P433/2 was highly virulent for mutants of *E. coli* P433 resistant to phage P433/1; phage P433/2 was completely inactive on *E. coli* P433.” Thus, they were able to explicitly quantify their concept of phage antibacterial virulence as “Smallest no. of phage particles required to lyse the *E. coli* culture” (p. 2662). Phage antibacterial virulence assays consequently can be viewed as quantifiable extensions of phage turbidity reduction assays that involve varying input ratios of phages to bacteria, an approach further elaborated upon with modern methods (Section 4.2).

### 4.2. Optical Density-Based Phage Virulence Determination

The basic means of assessing phage antibacterial virulence is to mix phages starting with progressively lower titers while holding bacterial concentrations constant and/or starting bacteria at progressively higher concentrations while holding the starting phage titers constant [48]. Often, the phages are started at a somewhat low multiplicity so as to require multiple rounds of infection and lysis to infect a majority of the bacteria present. Those phages that are able to lyse cultures with the largest gap between starting phage and bacterial concentrations are considered to be the most virulent. This is evident especially in the data of Storms et al. [50]. Varying starting phage multiplicities, they identified phage T5 with its slower impact as having a lower virulence than phage T7, which displays an earlier as well as overall greater impact.

Though seemingly straightforward, there nonetheless are limitations to typical optical density-based approaches to determining phage virulence. The first is the noted reestablishment of culture turbidities due to bacterial evolution of phage resistance. That issue, though, can be avoided through the use of kinetic rather than endpoint determinations, so that earlier growth of cultures can be more readily distinguished from later grow back of phage-resistant bacteria (Section 2.2.2) [17], and particularly by ceasing assays prior to the associated grow back [50].

The second issue stems from emphasis on phage-free bacterial control curves, which visually can deemphasize phage impacts on bacterial turbidities [16]. That emphasis on phage-free controls can be inherent to AUC determinations (Section 2.2), particularly if phage antibacterial virulence is being determined by comparing optical density outcomes with phages to outcomes without phages over long incubations. As examples, see Xie et al.’s [48] “liquid assay score”, Storms et al.’s [50] “local virulence”, Konopacki et al.’s [51] “PhageScore”, Ceballos and Stacy’s [62] “inhibition”, and Paranos et al.’s [56] “growth inhibition”. It is typical when this is performed for lysis-profile graphs to possess *y*-axis ranges that emphasize bacterial maximum turbidities over the details of phage impacts on that population growth. Note, though, that this emphasis on bacterial growth can be reduced visually by log-transforming *y* axes. That allows better emphasis on lower turbidities where direct phage impact on culture turbidities often takes place. For example, see the stretching of the phage T3 curve in Figure 2’s panel A vs. panel B.

The third issue results from reduced emphasis on phage bactericidal activity vs. bacteriolytic activity. That is, otherwise highly virulent phages—virulent in the sense of being able to relatively quickly kill a population of broth-growing bacteria—do not necessarily also rapidly lyse those bacteria. In other words, ideally with optical density-based virulence assays, both clearing of cultures and killing of bacteria will mostly coincide temporally. Nonetheless, that is not always the case, as the following sections (Section 4.3 and Section 4.4) consider.

### 4.3. False Lower Virulence with Lysis Inhibition

Lysis inhibition [15,16,17] is a phenotype seen with an important subset of phage types [17,85,86,87,88,89]. It involves a latent-period extension and burst size enhancement that is induced by the adsorption of phages, such as phage T4, to already phage-infected bacteria. When starting with lower phage multiplicities, these same phages instead display what can be described as a rapid lysis, which is equivalent to a typical phage latent period and typical phage burst size. As phage populations increase in number, however, a transition occurs as phages come to outnumber bacteria. At this point, phage multiplicities come to exceed 1—meaning that excess numbers of phage virions are present—and the result can be induction of lysis inhibition across cultures. Thus, fast phage population growth, prior to display of lysis inhibition by a majority of phage infections, is followed by slow bacterial lysis, after display of lysis inhibition also by a majority of phage infections.

**Figure 3:**
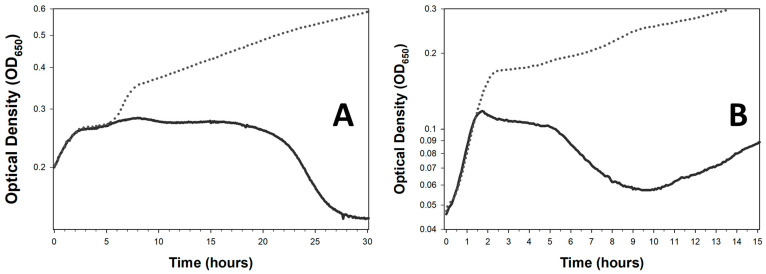
Examples of extended lysis profiles. Both panels involve phage T6 (solid lines), which classically is known to display lysis inhibition [39,41,85,92], with *E. coli* B growing alone (dotted lines). (**A**): There exist at least two difficulties toward virulence interpretation with this experiment. The first issue is just how long it takes until the observed turbidity decline, which occurs roughly 1 day into the experiment. The second issue is that the start of deviation, here occurring around six hours, does not necessarily coincide with phage T6 infection of a majority of bacteria present, which could have occurred as early as sometime after two hours. Nonetheless, the phage presumably did succeed in infecting a majority of the bacteria present and did succeed eventually in clearing the culture, this despite the relatively high peak turbidity reached by the T6 curve (10^8^ bacteria/mL corresponds to an optical density of about 0.1). Lysis in turn appears to be complete prior to 30 h into the experiment. The shape of the *E. coli* B curve is typical for this growth medium (Mueller–Hinton cation-adjusted) and experimental protocol (the latter the same as for Figure 1 and Figure 2). (**B**): This is an equivalent experiment except initiated at a lower optical density and run on a different day. In this case, the start of deviation is unambiguous, implying adsorption of a majority of bacteria especially given the subsequent decline in culture turbidity (the consensus AI-generated timing of the start of deviation is 88 ± 3.7 min with a range of calculated values from 80 to 92 min, which by observation appears to be accurate; S.T.A., unpublished methods). The start of turbidity decline is less definitive. This is in part because of the scale of the *x* and *y* axes—which inherently result visually in a more gradual decline—but also because of the recovery in turbidity that presumably is a consequence of prior bacterial mutation to phage resistance (around 9 to 10 h into the panel-B experiment). Certainly the turbidity decline is not as abrupt as that seen with phages T3 and K3 or even LZ9 in Figure 2.

As a consequence, phages that can display lysis inhibition possess a dual nature—no lysis delay that is followed by lysis delay. The result is that approaches used by Xie et al. [48] and Storms et al. [50] to determine phage antibacterial virulence can be confounded. This is due to the longer time frames required for those phages to substantially reduce the turbidity of a bacterial culture (Figure 1, Figure 2 and Figure 3, and the phage T4 curves of Storms et al. [50]). Such cultures may not appear to lyse much at all over a given time frame (Figure 3)—particularly given insufficiently long incubations—or instead may not lyse as fast as an equivalent phage not displaying lysis inhibition [17,40,90,91] (compare in Figure 2 the T3 curve with all of the other curves). The infection of a majority of bacteria present, however, may still be relatively rapidly accomplished, independent of the subsequent occurrence of lysis inhibition. It is just that once a large majority of bacteria are phage-infected, and thereby those bacteria are no longer able to replicate, these phage infections can delay their lysis.

Lysis inhibition thus can give a false impression of lower phage virulence. Specifically, lysis seems to occur many hours after deviation, the latter time point being when presumably all of the phage-sensitive bacteria in a culture have become phage-infected. Thus, the timing of lysis as a measure of phage antibacterial virulence can be misleading in the case of lysis inhibition if serving as a correlate for the timing of phage bactericidal activity.

Overall, being able to rapidly lyse bacterial cultures starting with fewer phages could be useful toward success in phage therapy and could represent a reasonable definition of higher phage antibacterial virulence. Nonetheless, optical density determinations can falsely identify bactericidally higher-virulence phages as having a lower virulence if those phages otherwise can display lysis inhibition. These same lysis-inhibiting phages also often display smaller plaques [15,93], and plaque size too can serve as a surrogate for more effective phage growth parameters [65,94,95].

### 4.4. Lysis Delay Without Strictly Lysis Inhibition

**Figure 4:**
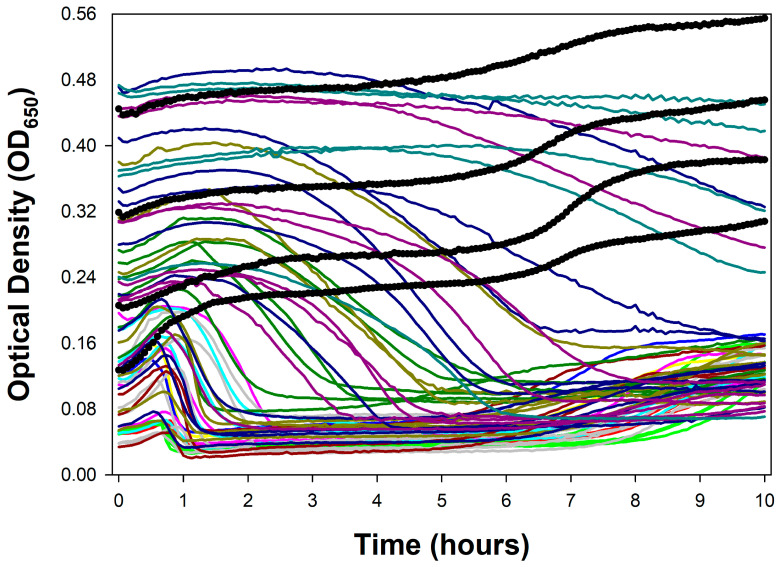
Variation in the timing of phage-induced bacterial lysis as a function of different starting culture turbidities. Starting multiplicities for phage T3—which is not thought to be capable of lysis inhibition sensu stricto [85]—were somewhat less than 1, resulting in initial rises in culture turbidities prior to cultures reaching their OD_max_. Different biological replicates are indicated by using different colors. Example bacteria-only curves are shown in black with closed circles as symbols. While the molecular mechanisms underlying these latent period extensions were not explored, the key observation is that despite the delay in lysis, an eventual turbidity decline for most of the presented curves appears to have occurred. That turbidity decline is likely consistent with phages lytically infecting a substantial fraction of bacteria present and suggests a reasonably high level of antibacterial virulence. This is even though actual lysis is delayed at these higher optical densities, as would suggest a much lower virulence if tallied according to either lysis-timing or AUC determinations. Note that otherwise the expected latent period of phage T3 is around 13 min [100], as presumably corresponding to the lysis seen occurring in the figure around the 1 h mark at lower starting culture optical densities (that hour takes into account not just the phage T3 latent period but also prior phage T3 increases in population size). That latent period appears to have been extended as seen especially above an optical density of 0.2 and certainly by the indicated 0.24 optical density (where 0.1 corresponds approximately to 10^8^ bacteria/mL). These presumed extended infection times manifest especially as much slower rates of culture turbidity declines. An equivalent figure but based instead on phage T1 can be found in [99]. This effect also has been seen with phage T7 (S.T.A., unpublished observation) and with the phage T4 *r48* mutant [17]. None of those phages, T1, T3, T7, or phage T4 *r48*, are expected to display lysis inhibition, sensu stricto [41,85,90]. These experiments otherwise were run equivalently to those presented in Figure 3.

Lysis inhibition is induced by virion adsorption of an already lytically infected bacterium, such as a phage T4 virion adsorbing a phage T4-infected cell. A seemingly similar phenotype is seen with various phages that appear to be able to display longer latent periods, as determined by optical density means, when infecting bacterial cultures that have grown to higher turbidities, yet display rapid lysis at lower turbidities [16,18,19,45,71,72,96,97,98]. It is uncertain, however, to what extent this phenomenon is related to lysis inhibition, as at least two obligately lytic phages that are thought to not be able to display lysis inhibition—phage T1 and the phage T4 *r48* mutant [85,90]—can clearly show this phenotype [17,99], and see also Figure 4 (phage T3 [85]). Also contrasting with lysis inhibition, this delay, as noted, is only seen at higher bacterial concentrations, whereas lysis inhibition can take place even at lower bacterial concentrations (e.g., Figure 3B). Nonetheless, this seemingly non-lysis inhibition delay, like lysis inhibition sensu stricto, can result in an impression of lower phage virulence. Delayed bacteriolytic activity, however, does not necessarily imply delayed bactericidal activity once phages have infected a majority of bacteria present.

### 4.5. Considering More than Just Declines in Optical Density

The above discussions suggest that there could be utility—for phage therapy purposes—in not discarding at least some phages due simply to their displaying (1) lysis delays, (2) seemingly poor antibacterial virulence when employing AUC-based optical-density determinations, and/or (3) small plaques. Rather, those phages might be tested further using alternative measures of especially phage bactericidal virulence, such as the timing of the start of deviation (Section 2.1), or non-optical density approaches (Section 4.5.1 and Section 5). This is lest otherwise good phages be gratuitously discarded. Nonetheless, there may be more to successfully assigning phage antibacterial virulence than just choosing which optical-density metric to use, such as AUC vs. OD_max_ vs. deviation vs. bacterial viability, etc.

#### 4.5.1. Alternative Approaches and Alternative Considerations

Discarding possible higher-virulence but slower-lysing phages—ones that are potentially useful therapeutically but less obviously so—can be avoided by plating for uninfected bacteria (Section 5) rather than characterizing phage virulence based solely on culture turbidity determinations. That is, performing true bacterial reduction assays. Doing that, however, tends to be more laborious than employing optical density-based methods. Plating for phage-uninfected bacteria also should be performed prior to the likely regrowth of cultures due to bacterial evolution of phage resistance. Such additional effort may be preferable to labeling phages as having low bactericidal virulence when that is not actually the case, and this is particularly so if alternative phages are difficult to obtain [101,102]. Otherwise, though less well-developed as a phage virulence indicator, is the above-noted start-of-deviation metric (Section 2.1; Figure 1, Figure 2 and Figure 3).

A perhaps equally important consideration is whether phage antibacterial virulence as typically determined—where rates of phage population growth from low starting multiplicities play a substantial role—is always relevant to phage therapy success. Three distinct phage properties help to frame this question: reproductive ability (Section 4.5.2), bactericidal activity (Section 4.5.3), and bacteriolytic activity (Section 4.5.4).

#### 4.5.2. Phage Population Growth

A phage’s ability to produce new virions while infecting a targeted bacterium should matter to phage therapy outcomes only to the extent that phage replication in situ is needed for phage therapy success (so-called phage-therapy active treatment [5,21,22]). If bacteria are readily accessible to phages, and phages can be applied to those bacteria in high enough numbers, then it is possible that this aspect of phage antibacterial virulence—a phage’s ability to rapidly or substantially grow its population—in at least some cases may be less important. That leaves then bactericidal and bacteriolytic activities.

#### 4.5.3. Focusing on Killing Bacteria

Ultimately, a primary goal of phage therapy should be giving rise to the killing of targeted bacteria, as also is the primary goal of antibiotic therapies. In terms of optical density-based virulence assays, presumed bactericidal activity could be assessed as a distinct metric by employing higher starting phage multiplicities and titers, such as 5 × 10^8^ bacteria/mL and a multiplicity of at least 5. Should phage application result in a substantial number of bacteria no longer being able to replicate, then that may be inferred based solely on the noted start of deviation of the optical density of phage-containing cultures away from that of the phage-free control. Interference with bacterial replication, even if only temporary, should result in a deviation of phage-containing curves from those of phage-less controls.

Adding large numbers of phages thus ought to abruptly stop culture turbidities from rising, and this is so even if the phages are intentionally non-replicative or non-lytic [103,104,105]. Such an optical density-based assay explicitly has been used to assess the killing potential of a genetically engineered, non-replicating, and non-lytic but bactericidal filamentous phage, as corroborated in terms of plating for bacterial viable counts [106]. Equivalently, though without phage involvement, such optical density-based killing assays can be used to assess the antibacterial impact of induced toxin expression by plasmids [107], also as corroborated there in terms of bacterial viable counts.

Furthermore, bactericidal activity without lysis may be distinguished from phage-induced lysis from without of bacteria—the former shows an absence of substantial, sustained turbidity decline while the latter is indicated by a rapid turbidity decline that starts relatively soon after phage addition [17,108]. If in doubt, however, one can adjust phage titers downward until one observes a cessation of optical-density rise without substantial optical density decline.

This same assay can be performed using bacteria that have been grown to higher concentrations such as 2 × 10^8^ or 4 × 10^8^ bacteria/mL—while maintaining the same higher phage multiplicity—to indicate the potential for phage bactericidal activity against physiologically older bacteria. This is striving toward what may be achieved with plating methods, but instead using an optical-density determination: deviation as a stand-in for declining colony-forming units.

#### 4.5.4. Phage-Mediated Bacterial Lysis

What about the clearing of cultures? For that to occur, then a phage must not only be bactericidal but also bacteriolytic. To the extent that bacteriolysis is required for phage therapy success, then this factor certainly would be relevant, though some phages likely are more reliable at accomplishing this lysis, particularly at higher bacterial concentrations, than others (S.T.A., unpublished observation). This issue too can be tested using optical density means, by again using high phage multiplicities and high phage titers, plus varying the extent that cultures have been grown prior to adding phages.

It is entirely possible that phages which are effective at lysing late-log-phase bacteria, or alternatively, at actively penetrating into bacterial biofilms [5,22], could be more useful for certain phage therapy purposes than phages that are especially good at clearing broth cultures starting with small phage numbers. The latter, though, may also correlate with an ability to lyse later-log-phase bacteria. Alternatively, minimizing bacterial lysis can be associated with reduced release of toxins such as endotoxin [109,110]. This suggests that, instead of being a defect, phage display of slower lysis could actually be beneficial therapeutically, at least so long as this does not correlate with delayed phage infection of a majority of targeted bacteria.

#### 4.5.5. Treatment Requirements Should Dictate Phage Choice

In short, reliance on a combination of time and lysis as measures of phage antibacterial virulence might be limiting as estimators of actual phage antibacterial activity during phage therapy treatments. What could be just as important is first determining whether a given therapeutic treatment requires phage replication and/or bacterial lysis in situ to be effective, and then employing phage virulence assays that more effectively assess those properties. Depending on treatment circumstances, therefore, what are now standard phage antibacterial virulence assays [48,50,51,56] may not always be able to indicate which phages might be most useful for a given phage-therapy purpose.

## 5. Time-Kill Curves

Related to phage antibacterial virulence determinations are time-kill assays. Both score a greater antibacterial impact as either sooner or more-complete decreases in bacterial presence. With AUC-based virulence determinations, this is a consequence of faster turbidity reductions that result in curves dropping more quickly, ideally to extinction (e.g., Figure 2, phage T3 curve). Time-kill assays likewise are based on the timing and degree of this drop toward bacterial extinction. The latter description can be confusing, however, as some publications present time-kill analyses based on bacterial reductions recorded after a single set period of time, such as 24 h, even if time points were taken earlier [111,112,113,114,115,116,117]. Recall in particular concerns over bacterial evolution of phage resistance given end-point determinations, particularly following extended incubations [17]. Ideally, however, what should be taken into account is the actual timing of drops in bacterial viability, resulting in actual time-kill curves, e.g., [118,119,120,121].

### 5.1. Traditional Time-Kill Assays Make More Sense for Antibiotics than for Phages

In her CRC Press review chapter, Verma [122] noted (emphasis hers; pp. 286–287) that, “The killing effect of an antimicrobial agent can be expressed as the rate of killing by a fixed concentration of drug under controlled conditions. This rate is determined by measuring the number of viable bacteria at various time intervals. The resulting graphic depiction is known as a *time-kill curve*. … killing curves measure changes over time. … A steep negative slope of the curve indicates a faster rate of decline of survivors.”

Though the resulting kinetic assays should generally be of utility for characterizing antibiotics, I will argue here that—as typically implemented—time-kill curves may not be ideal for precisely gauging the timing of phage impacts on broth bacterial cultures. Rather, phages in most cases are expected to quickly transition cultures from most-bacteria-are-still-alive to most-bacteria-are-now-dead, during broth-based assays. The timing of that transition cannot be captured using endpoint data, nor even using kinetic assays with, e.g., time intervals in the range of one hour or longer. Indeed, Verma [122] notes further that (p. 290), “For antimicrobial agents that exhibit concentration-dependent killing, organisms may be killed faster at concentrations greater than the MIC, requiring sampling at shorter time intervals.”

This temporal distinction between phage-mediated and typical antibiotic-mediated bacterial killing suggests a utility for using optical density-based methods for time-kill analyses. This is versus “cumbersome and laborious” (p. 290) [122] bacterial viable count determinations traditionally used to generate time-kill curves. But this is only an option if optical density assays are performed in such a way that the phage impact on bacterial viability is emphasized; also, so long as time points are taken over short intervals such as somewhat more than one per hour—shorter sampling intervals can be seen in Figure 1, Figure 2, Figure 3 and Figure 4, where 4 min time points were used. We have already considered many of these optical density issues in the guise of phage antibacterial virulence assays (Section 4), but these are further elaborated upon in the following sections.

### 5.2. Phage Time-Kill Assays Are Phage Antibacterial Virulence Assays

Phage-mediated time-kill assays have been conducted, or at least claimed to have been conducted (Appendix A), in a number of studies measuring phage-mediated changes in culture optical density [123,124,125,126,127,128,129,130,131,132,133,134,135,136,137,138,139]. Thus, for example, “time-kill kinetics assay through kinetic optical density measurements” (quoting [140] as referring to [141]; see also Appendix A.18) or “time-kill assays, where the kinetics of the phage-induced lysis is assessed by measuring the optical density” [142]. See Figure 4 for illustration of how the rapidity of phage-mediated changes in culture optical density can vary with starting bacterial densities.

But how does that differ from phage antibacterial virulence assays as considered in Section 4? Indeed, when conducted using optical density-based methods, there actually is little difference between phage antibacterial virulence assays and time-kill assays. This especially holds if one emphasizes the timing of bacterial killing for time-kill assays, though with optical density-based assays that bacterial killing must be inferred rather than being directly measurable as viable counts.

There is good reason to emphasize the timing of the transition from predominant culture viability to predominant culture inviability, rather than emphasizing the rate of bacterial killing or the degree of that killing after a specified interval of time. This is because for phages, as noted (Section 5.1), once substantial bacterial killing has begun—as a consequence of phage adsorption of bacteria—that killing is expected to proceed rapidly, particularly for broth-based assays. This is whether phages are applied at a higher multiplicity or instead first have to grow their populations to those multiplicities (the latter being auto dosing). In addition, emphasizing the timing of killing rather than using, e.g., AUC determinations, should avoid the complication of culture regrowth due to bacterial evolution of phage resistance.

Thus, phages generally kill bacteria more quickly than antibiotics do, but do so only once sufficient numbers of phages are present. Time-kill assays for phages consequently should emphasize determining the timing especially of that killing. Taking into account the timing of phage-mediated bacterial killing therefore should at least be considered as well with phage antibacterial-virulence assays (Section 4.5). For optical density-based assays, likely the best correlate we have to determining the timing of bacterial genetic death, indicated as an inability to form colonies, is the point of deviation.

### 5.3. The Size of Time Intervals Does Matter

Time-kill assays for antibiotics in many publications are performed using 1- or more-hour time intervals, e.g., [119,143,144,145,146]. This long delay between data points is presumably due to how laborious plating-based studies can be, but also because it often takes substantial amounts of time for even bactericidal antibiotics to kill bacteria. For phages, by contrast, 5 min or shorter time intervals would be ideal, though practically speaking such frequent sampling can only be accomplished over longer time periods using optical density-based methods (Figure 1, Figure 2, Figure 3 and Figure 4).

Of interest, endolysins [147] also can display rapid bacterial killing. They therefore also require shorter time intervals during time-kill assays to fully capture this killing [148], and also are commonly assessed by optical density means, e.g., [149,150,151,152]. However, as endolysins are simply enzymes, they lack the phage potential for auto dosing. As a result, substantial lysis should begin very soon after endolysin application, if substantial lysis is going to occur at all. That should make plating-based assays more practical for characterizing endolysin treatments than for low-multiplicity assays using phages. This is even though multiple time points would still need to be taken over short intervals to capture the timing of endolysin-mediated killing, i.e., as a time-kill curve.

Thus, we can distinguish among antibacterial agents in terms of whether their bactericidal activity is fast (endolysins, phages) or slow (many antibiotics). Among fast-acting agents, the timing of bactericidal activity is either easily captured by standard CFU-based time-kill curves (endolysins) or not easily captured (phages), the latter due to difficulties in predicting rates of phage population growth de novo. The less certain we can be about when time points should be taken (phages but less so endolysins or antibiotics), then the greater the utility of automating data collection, such as by using optical density-based measurements. Furthermore, the more closely spaced those measurements are made, then the higher the precision of any resulting determination. Thus, for phage-based time kill assays, size does matter, but here it is increasing the total number of data points taken per unit time that can be crucial, rather than increasing the intervals between time points.

### 5.4. Emphasizing “Kill”, and Deviation

Section 4 questions how effective AUC-based phage antibacterial virulence assays are at distinguishing among phages in terms of the timing of their bactericidal activity. The problem in particular is that there is a disconnect between the occurrence of bacterial lysis and the likely occurrence of bacterial killing. Plating-based time-kill assays, by contrast, are consistently based on measurements of bacterial viability.

For many phages, especially when treating mid-log-phase bacteria, this distinction between lysis timing and bacterial killing matters less, given relatively short latent periods. For example, latent periods are 13 to 25 min for phages T1 through T7, except for phage T5 with a 40 min latent period [100]. However, for phages that inherently can display very long latent periods during optical density-based assays (Section 4.3; Figure 1, Figure 2 and Figure 3), or which can display longer latent periods when infecting later-log-phase bacteria (Section 4.4; Figure 3 and Figure 4), the temporal difference between bacterial killing and bacterial lysis can be hours. This creates a problem for optical density-based time-kill assays, particularly when treating physiologically older bacteria—and physiologically older bacteria certainly are not irrelevant when treating especially chronic bacterial infections using phages [153].

An important aspect of time-kill assays is to explore, in vitro, the antibacterial potential of treatments consisting of combinations of more than one antibiotic (e.g., [143,144,145,154,155]), more than one type of phage [115,156,157] (known as phage cocktails [6,158,159,160,161,162,163,164,165,166,167,168,169,170,171,172,173]), or combinations of phages and antibiotics [111,112,113,114,115,116,123,128,132,133,136,156,174,175,176,177,178,179,180,181,182,183,184,185,186,187,188,189,190,191]. This typically is undertaken to look for instances of synergistic or antagonistic interactions between agents [122], though when assaying for synergistic interactions between phages and antibiotics, typically sub-inhibitory concentrations of antibiotics are employed. When tested individually for bacterial killing, however, inhibitory concentrations of antibiotics should be used, lest there be an absence of actual bacterial killing. Indeed, inhibitory concentrations of antibiotics are generally used clinically.

Unlike antibiotics at standard inhibitory concentrations, inhibitory concentrations of treatment phages are expected to be both rapidly and obligately bactericidal. Consequently, if sufficient numbers of phages are added, then a bacterial culture should be quickly reduced to just phage-resistant bacteria. The utility of measuring time-kill kinetics with phages therefore should stem from assessing the impact of lower phage titers—which without auto dosing are sub-inhibitory—rather than rapidly bactericidal higher phage titers. Thus, phage time-kill assays in effect are even more similar to typical phage-virulence assays; they incorporate not just phage antibacterial activity but also phage population growth. In contrast, for antibiotics or purified phage endolysins (Section 5.1), time-kill assays of course cannot involve this population-growth component.

Taken together, optical density-based time-kill assays, especially with their bacteria-killing emphasis, therefore should not be primarily driven by the occurrence of bacteriolysis. They instead should employ the start of deviation, and do so particularly starting with phage multiplicities of less than one (Section 4.5)—this is at least to the extent that the start of deviation more readily correlates with bacterial killing than the timing of culture-wide bacterial lysis. Thus, time-kill assays as traditionally administered can be viewed as equivalent to phage antibacterial virulence assays, but with a focus especially on the occurrence of phage-mediated bacterial killing. This is rather than an emphasis primarily on reductions in bacterial biomass.

### 5.5. What if Your Cultures Do Not Lyse?

As a caveat, if phage adsorption is not rapid, then phage-mediated killing also may not be rapid. This may be to a point where bacterial *growth* reduction is observed (Section 3.2) rather than actual bacterial reduction (Section 3.1). If slow adsorption is suspected, rather than just delayed lysis (Section 4.3 and Section 4.4), then plating for bacterial viable counts soon after the start of deviation may be useful. This is to distinguish substantial phage adsorption but slow lysis from insubstantial phage adsorption and therefore insubstantial bacterial killing. Here “soon” means after tens of minutes rather than after many hours. If neither turbidity nor viable counts are substantially declining—despite the occurrence of visible deviation—then for obligately lytic phages one should suspect very slow phage adsorption.

## 6. Conclusions

My general conclusion is that optical density-based phage antibacterial virulence assays ideally will strive to approximate the results that can be attained using time-kill assays that are based on plating. To the extent that the timing of the start of deviation can achieve that result—time of start of deviation ≈ time of start of substantial bacterial dying (S.T.A., unpublished observation)—then that likely could serve as a superior metric for optical density-based phage-virulence assessments.

Both of those conclusions—a utility for time-based metrics and a superiority for actual time-kill assays—nonetheless assume that successful phage therapy will by necessity rely upon in situ phage population growth. That, however, is not necessarily always the case, suggesting that simply demonstrating deviation upon higher multiplicity phage addition could serve in many cases as an adequate indication of a phage’s suitability for treatment. This is the difference between therapeutic requirements of active vs. passive treatments, with traditional time-kill curves serving as useful in vitro models of active phage treatments whereas high multiplicity treatments instead will model passive treatments.

Possibly as important, in vitro treatment conditions need to resemble those during clinical treatments, and in many cases that should mean that deviation needs to be achieved even when employing target bacteria that have grown well past mid-log phase. That statement, though, comes with a caveat. Namely, this likely does not include actual stationary phase since without observable bacterial growth, there also may be no deviation from ongoing increases in culture optical densities (and likely as well, no phage-induced bacterial lysis). Similarly, in phage-antibiotic combination treatments, if co-applied bacteriostatic antibiotics prevent bacterial growth, there may be no potential for phage-mediated deviation either, unless sub-inhibitory antibiotic concentrations are being used instead.

In any case, the start of deviation is the initial optical density difference between a phage-treated culture and a phage-less culture, where the latter is continuing to grow in turbidity. Computational methods for objectively determining this point from relatively noisy optical-density data are currently under development.

How relevant phage-induced bacterial lysis is to phage therapy success remains an open question, versus simply infection by phages and consequent bacterial killing (loss of colony-forming ability). After all, not all bactericidal antibiotics are also bacteriolytic. Such lysis, however, could be relevant especially for phage treatment of bacterial biofilms, to the extent that ongoing bacterial lysis is required for phage penetration into biofilms, to beneath surface-associated bacteria (so-called active penetration [5,22]). Thus, a phage that can successfully kill and then lyse bacteria, especially physiologically older bacteria, could in some cases serve as a superior treatment phage. And the same phage might display that superiority independent of rates of phage population growth, where the latter property appears to be central to standard AUC-based determinations of phage antibacterial virulence or optical density-based time-kill assays as currently practiced.

Optical density-based approaches to characterizing bacteriophages for phage therapy purposes thus

- Need to reflect the physiologies of the bacterial infections to be treated;
- Need to better focus on those phage properties that are likely to be most relevant to phage therapy successes (whether bactericidal, bacteriolytic, or reproductive); and
- Need to be less focused on bacterial evolution of phage resistance (unless that is a study’s goal), as measuring phage killing of susceptible bacteria and tracking resistance evolution are distinct research questions requiring methodologically separate approaches.

Ultimately, demonstration of some degree of bacterial reduction (strong bactericidal activity) also should be viewed as superior to merely bacterial growth reduction (minimal turbidity reduction).

To summarize, (1) these three assays—turbidity reduction, antibacterial virulence, and time-kill—are related means of measuring phage impact on bacterial cultures, though with slightly different emphases. (2) Of the various ways that this impact can be characterized for phage therapy purposes, bacterial killing should be the most useful, as emphasized by the importance of time-kill curves in characterizing antibiotics. (3) Optical density approaches to phage characterization can be more convenient than plating-based assays, including for adapting time-kill assays for phage use. (4) Deviation appears to represent a superior correlate to phage bactericidal activity than, for example, culture turbidity reduction. Lastly, (5) a phage that fails to ever display any deviation likely can be rejected as unsuitable for treating that bacterial strain under the conditions tested.

## Data Availability

Data for the original contributions presented in this study are included in the file, Supplementary Materials S1.

## Supplementary Materials

The following supporting information can be downloaded at: https://www.mdpi.com/article/10.3390/v18010097/s1. Data for the original contributions presented in this study are included in the file, Supplementary Materials S1. Further inquiries can be directed to the author.

## Appendix A. Optical Density Time-Kill Assays: Critical Assessment

A number of studies present what are described as time-kill assays (or equivalent terminology) using optical density measurements rather than reductions in bacterial viable counts. In this appendix I consider each of these studies in terms of their utility in providing time-kill information. The eventual goal, though not fully addressed in this appendix, is to improve the utility of time-kill assessments of phage therapy purposes.

Overall, and to the best of my knowledge, a label of “time-kill” (or “time-killing”) has been used for optical density-based assays in a total of 17 studies. For eight of those studies, true time-kill information can be inferred [126,128,129,132,133,134,135,136]. Seven others are more suppression of bacterial evolution of phage resistance [21] assays [123,124,127,131,137,138,139], and for two time-kill information may be present but is highly imprecise due to a use of two-hour intervals between time points [125,130]. As follows I summarize those studies. Also discussed is Ferry et al. [141] as cited by Perlemoine et al. [140].

A general observation concerns the appropriateness of different time intervals during assays. A suggestion is that time intervals should not be longer than measured phage latent periods as determined separately unless there is a compelling reason to use longer intervals. Indeed, time intervals in the range of five-min or less in particular take full advantage of optical-density approaches to time-kill determination. Alternatively, one-hour time intervals provide poor resolution while even longer intervals such as two hours can make it very difficult to distinguish between different curves in terms of time-kill kinetics.

### Appendix A.1. Lin et al., 2018 [123] (Resistance Suppression Assay)

Lin et al. [123] explored the impact of phage PEV20 in combination with various antibiotics on three strains of *P. aeruginosa*. They used four time points, at 0, 4, 8, and 24 h, and 10^6^ CFUs/mL early log phase bacteria. For phage treatments alone, optical density remained at zero for the 0 4, and 8 h, providing effectively no time-kill information for two of the bacterial strains tested. For a third strain, there didn’t appear to be any indication of bacterial reduction by 4 h, but there was bacterial growth reduction by 8 h.

### Appendix A.2. Horváth et al., 2020 [124] (Resistance Suppression Assay)

Horváth et al., [124] resuspended an overnight culture of *Klebsiella pneumoniae* to 10^5^ CFUs/mL, which barely increased in turbidity over two hours. Phage vB_KpnS_Kp13 was added with multiplicities ranging over ten-fold increments from 0.0001 to 100, which is a calculated 10^1^ to 10^7^ phages/mL at the start of experiments. As expected given the low starting bacterial concentration, no turbidity reduction is observed for any of the phage titers added. By 10 h and later, a slight recovery in turbidity is observed for some of the curves, suggesting growth of phage-resistant bacteria. Reported only was that bacterial growth was inhibited over a 24-h interval, which suggests that the assay was used to test the ability of this phage to suppress bacterial evolution of phage resistance [21] rather than time until bacterial killing.

### Appendix A.3. Chaudhary et al., 2021 [125] (Imprecise Time-Kill Curves)

Chaudhary et al. [125] tested phage RDN37 against an *E. coli* strain. Time points were taken every two hours and by two hours all three starting phage multiplicities (0.01, 0.1, and 1) may have achieved deviation. For all three phage multiplicities, a turbidity reduction relative to the 2-h time point is apparent by 4 h. Due to the size of intervals between time points, minimal information concerning the timing of phage-associated bacteria killing is obtainable. The authors cite Huang et al. [192] for their time-kill protocol, whereas Huang et al. describe their equivalent experiments as “lysis ability” assays.

### Appendix A.4. Naknaen et al., 2021 [126] (Time-Kill Curve)

Naknaen et al., [126] used a time-kill curve to study the impact of cyanophage PhiMa05 on *Microcystis* SG03 with and without bacterial aggregation. Without aggregations, multiplicities ranged from 0.001 to 100, incremented by powers of 10 (OD_680_ started around 0.05, corresponding to 10^6^ CFUs/mL). For the two highest multiplicities, deviation occurred by 3 days (all intervals were in days) with turbidity reduction clearly present at that point for the highest multiplicity. For a multiplicity of 1, deviation was by 4 days and lysis by 5. For a multiplicity of 0.1, deviation likely occurred by day 4 with lysis obvious by day 6. The two lowest multiplicities showed deviation by day 5, and lysis also by day 6. For aggregated cells, OD_680_ started instead around 0.1. Indicated multiplicities were 0.1 through 100, also incremented by powers of 10. Deviation of varying degrees seems to be seen by three days for all four multiplicities. Lysis appears to have occurred by day 4 for the highest multiplicity, day 6 for the lowest, and day 5 for the two middle multiplicities (based on 1-day time intervals).

A one-step growth curve [193,194,195] was performed indicating a latent period (as constant period) between 1 and 2 days long. As a result, the 1-day intervals between time points are shorter than the phage’s latent period. By contrast, Chaudhary et al. [125] reported a latent period of 15 min, which is only 12.5% of their 2-h intervals between time points.

### Appendix A.5. Pertics et al., 2021 [127] (Resistance Suppression Assay)

Pertics et al., [127] used what they label as a time-kill assay to characterize what appears to have been “lytic ability” of phage B1 against a *Klebsiella pneumoniae* strain. Similar to Horváth et al., [124], they employed an overnight initiated culture consisting of 10^3^ CFUs/mL (though elsewhere they seem to claim that these are “exponential growth-phase cultures”). The resulting curves were also similar to those of Horváth et al., [124] except that the lag before a substantial increase in optical density for the phage-free control is even longer (though curves were graphed with 1-h time intervals, apparently 5-min time points were actually taken). Multiplicities ranged from 0.01 to 10,000. Some increase in turbidity is seen in some of the curves starting around the same time as the phage-less control, but those increases at most were only minimal over a 24-h period. An equivalent conclusion may be reached as for the Horváth et al., [124] experiment. From Pertics et al., this is, “The growth of the bacteria was repressed successfully by the phage at MOI between 0.01 and 10,000 in LB broth for 24 h.”

### Appendix A.6. Simon et al., 2021 [128] (Time-Kill Curve)

The time-kill assay presented by Simon et al. [128] takes place over a 16-h period with 20-min time intervals. For the presented figure, phage Sb-1 was added with a multiplicity of 0.1 to *Staphylococcus aureus* strain SA19. It appears to have taken between two and three hours for deviation to occur, assuming that phages were added at time = 0. If that deviation represents a true measure of phages infecting a majority of bacteria present, then that is an unexpectedly long interval. This, though, can perhaps be explained by the use of starting bacterial concentrations of 5 × 10^8^ CFUs/mL (colony-forming units; OD_590_ ≈ 0.5), which by observation appear to have been past the exponential portion of their growth curve. In addition, there is no indication of how many bacteria made up individual CFUs in this experiment. Peak optical density seems to have been reached by around 4 h after phage application (again, assuming the time of phage addition of zero). Substantial lysis didn’t begin until around 6 h into the experiment.

Deviation with oxacillin application alone occurred around the same time as for phage Sb-1, but deviation appears to have started perhaps slightly sooner when adding the phage in combination with the antibiotic. The latter suggests that killing could have occurred earlier with the combination therapy than with phage action alone, though the nature of the assay was such that this combined effect was dependent on the phage first replicating to a higher titer than what it started at.

This latter conclusion is possibly contradicted by equivalent AUC results obtained using a one-hundred-fold higher starting phage multiplicity. However, since deviation is not derivable from AUC values, it is difficult to gain much more understanding from that observation in terms of the timing of actual bacteria killing. Since cell wall synthesis-inhibiting antibiotics do not necessarily interfere with phage population growth prior to the occurrence of cell lysis [37], my guess is that the combination of phages and antibiotics resulted in sooner phage-induced bacterial lysis due to a destabilization of the bacterial cell wall by the oxacillin, but not necessarily also sooner phage-mediated genetic killing of bacteria. The latter conjecture, though, was not confirmed using plating-based measures. Some variation in deviation between phage isolates as well as between bacterial strains was observed.

### Appendix A.7. Whittard et al., 2021 [129] (Time-Kill Curve)

Whittard et al. [129] characterized multiple phage isolates against more than one *S. aureus* strain. Experiments were run using log-phase bacteria and initial phage multiplicities of 0.1. One-half-hour time intervals were employed. Of interest, some phages displayed a somewhat delayed lysis while infecting some but not all bacterial strains.

### Appendix A.8. Chaudhary et al., 2022 [130] (Imprecise Time-Kill Curves)

Chaudhary et al. [130] tested phage vB_EcoA_RDN8.1 against an *E. coli* strain initiated from at 10^8^ CFUs/mL from bacterial streak plate. Time points were taken every two hours and by two hours all three starting phage multiplicities (0.01, 0.1, and 1) had achieved deviation. For all three phage multiplicities, a turbidity reduction relative to the 2-h time point is apparent by 4 h. Especially with the lower starting phage multiplicities, substantial lysis is not apparent until the 6-h time point. Thus, overall, little information concerning the timing of phage-associated bacteria killing appears to be derivable from this experiment. The authors again cite Huang et al. [192] for their protocol.

### Appendix A.9. Schumann et al., 2022 [131] (Resistance Suppression Assay)

Schumann et al. [131] employed phage PAK_P1 against *P. aeruginosa* normoxia and hypoxia conditions. Multiplicities of 0.1, 1, and 10 were employed. Deviation appears to have occurred with both conditions in under two hours, but for all multiplicities this occurred before noticeable gains in optical density had occurred. That presumably is due to initiation of cultures with only 2 × 10^6^ CFUs/mL (growth-phase uncertain). Regrowth of the culture subsequently occurs, which is noticeable by around 12 h again for both conditions. The consistency of the timing of that grow back as well as the consistency of its rate leads to an assumption that the resistant-mutant is the same across the different multiplicity treatments. Given that it is impossible to tell with any precision when deviation occurred, I am hesitant to label this as an actual time-kill assay versus simply a kill assay with subsequent regrowth of presumably phage-resistant bacteria.

### Appendix A.10. Vashisth et al., 2022 [132] (Time-Kill Curve)

Vashisth et al. [132] treated *Acinetobacter baumannii*, *S. aureus*, and *Salmonella* Typhimurium with a variety of phages. One-hour time intervals were employed, which substantially limits time-kill precision. Nonetheless, clear starts of deviation are apparent. It is interesting, however, that under the conditions used, lysis as a turbidity decline in many, most, or all cases seems to have been substantially delayed (many hours). Assays were run for nine hours and remaining CFUs at that point were determined showing multiple-log reductions, though what fraction of surviving bacteria were phage-resistant is uncertain. Of interest, both optical density- and CFU-based analysis suggest antibiotic-mediated antagonism of phage antibacterial activities [37,38], which the authors point out and which was confirmed in many cases in terms of antibiotic impact on phage plaque size. Phage φST188 of *Salmonella*, however, appeared to display larger plaques in the presence of sub-inhibitory concentrations of clindamycin than without.

### Appendix A.11. Vashisth et al., 2022 [133] (Time-Kill Curve)

The Vashisth et al. [133] is similar to that of Vashisth et al. [132], here focusing on the impact of phage φAB182 of *A. baumannii*. This phage with a variety of antibiotics displays larger plaques in their vicinity, i.e., at sub-inhibitory antibiotic concentrations. This phage-antibiotic synergy result was corroborated in the presented time-kill assays. That synergistic impact on culture optical density is more easily quantified using peak culture turbidity or AUC determinations rather than deviation, where the latter provides distinctions that are difficult to visualize both as presented and given the use of one-hour time points.

### Appendix A.12. Abdelsattar et al., 2023 [134] (Time-Kill Curve)

Abdelsattar et al. [134] treated *Salmonella enterica* with phage ZCSE9. Though in their main text they don’t use the phrase time-kill (or, more specifically there, “time-killing”), their fourth figure appears to be their time-killing assay. This looks like a legitimate series of time-kill curves, ones in which deviations may be discerned and which was not extended long enough for the complication of growth of phage-resistant bacteria to become apparent. Phage multiplicities were also varied by powers of ten from 0.0001 to 10. Twenty-min time intervals were used.

### Appendix A.13. Li et al., 2023 [135] (Time-Kill Curve)

Li et al., [135] used phage K against *S. aureus* with starting phage multiplicities ranging from 0.000001 (10^−6^) to 0.1 study the kinetics of lysis though which they described as “time-killing profiles” (see their first figure). Time points were taken every hour with the 0.1 multiplicity showing a turbidity decline starting after 2 h, the 0.01 multiplicity showing this after 3 h, and the 0.001 multiplicity showing deviation by 3 h but only minimal subsequent lysis. The rest of the multiplicities showed deviation by 4 h but even less turbidity decline than the 0.001 multiplicity curve. Bacteria grown to a concentration of 10^8^ CFUs/mL appear to have been used to start assays, corresponding to a starting OD_600_ of 0.5. Going by the timing of deviation, this likely represented a legitimate time-kill assay, though with low resolution due to the long intervals between time points.

### Appendix A.14. Moryl et al., 2024 [136] (Time-Kill Curve)

Moryl et al. [136] characterized two phages of *Enterococcus faecalis*. Their time-kill curves in some cases indicated a clear start of deviation, though with time intervals of one hour. Stationary-phase cultures were used, diluted to 2 × 10^7^ CFUs/mL with phages added at a multiplicity of 0.1. Phage-antibiotic synergy (with ampicillin) appears to be observed as an earlier deviation, lower peak turbidity, and smaller AUC, all as judged visually.

### Appendix A.15. Sabri et al., 2024 [137] (Resistance Suppression Assay)

Sabri et al. [137] applied phage MATE 2 against *Xanthomonas campestris*. Starting bacterial concentrations varied from 10^2^ to 10^8^ CFUs/mL with time points taken every four hours with starting phage concentrations of 10^4^ or 10^8^ per ml. Generally, Deviation appears to have occurred later with lower starting bacterial concentrations but its timing is otherwise impossible to quantify with precision. Oddly, rather than presenting curves in terms of optical density (or absorbance), instead *y* axes are labeled as “Increase in absorbance”. The result is very little change in curves is observed until multiple hours into assays.

### Appendix A.16. Essam et al., 2025 [138] (Resistance Suppression Assay)

Essam et al. [138] treated *A. baumannii* cultures with phage ΦZC2 and ΦZC3 at multiplicities from 0.01 to 100. Experiments were performed in such a way—with one-hour time intervals and low starting bacterial concentrations (10^6^ CFUs/mL)—that no readily discernable substantial drops in turbidity were observed (though divergence was seen in all cases at the one-hour point). Increases in turbidity, however, were eventually seen starting 5–6 h into experiments.

### Appendix A.17. Urgeya et al., 2025 [139] (Resistance Suppression Assay)

Urgeya et al. [139] targeted various strains of *P. aeruginosa* (10^7^ CFUs/mL starting concentration) with six different phages. Phage multiplicities of 1, 10, and 100 were used. Except for one host strain (PA235) and one or two phages, which showed a lower impact with the lower multiplicity, results were similar. Deviation thus was mostly seen by two hours (two-hour time intervals were used), often with slight drops in optical density, and typically with some degree of subsequent culture grow back.

### Appendix A.18. Ferry et al., 2020 [141] by Way of Perlemoine et al., 2021 [140] (Explanation)

Perlemoine et al. [140] cites Ferry et al. [141] for use of optical density-based time-kill assays. However, Ferry et al. do not claim to have performed time-kill assays but rather describe ‘kinetic’ assays that they term ‘phagograms’ [196], used as a means of phage susceptibility testing. These assays employed low starting bacterial concentrations with phages added at multiplicities of 1, 10, or 100. While deviation timing varied with multiplicity, the authors’ interpretation was limited to susceptibility assessment (“…high susceptibility to at least two of the three phages at high MOI”), with no discussion of time-kill kinetics. The term ‘phagogram’ appears to be used analogously to ‘antibiogram’ for susceptibility testing [197,198], rather than indicating temporal kinetic analysis. Thus, this approach does not appear to constitute a time-kill assay despite being cited as such.
